# Energetics of the Glycosyl Transfer Reactions of Sucrose Phosphorylase

**DOI:** 10.1021/acs.biochem.3c00080

**Published:** 2023-05-30

**Authors:** Anisha Vyas, Bernd Nidetzky

**Affiliations:** † Institute of Biotechnology and Biochemical Engineering, Graz University of Technology, Petersgasse 12, Graz A-8010, Austria; ‡ Austrian Centre of Industrial Biotechnology, Krenngasse 37, Graz 8010, Austria

## Abstract

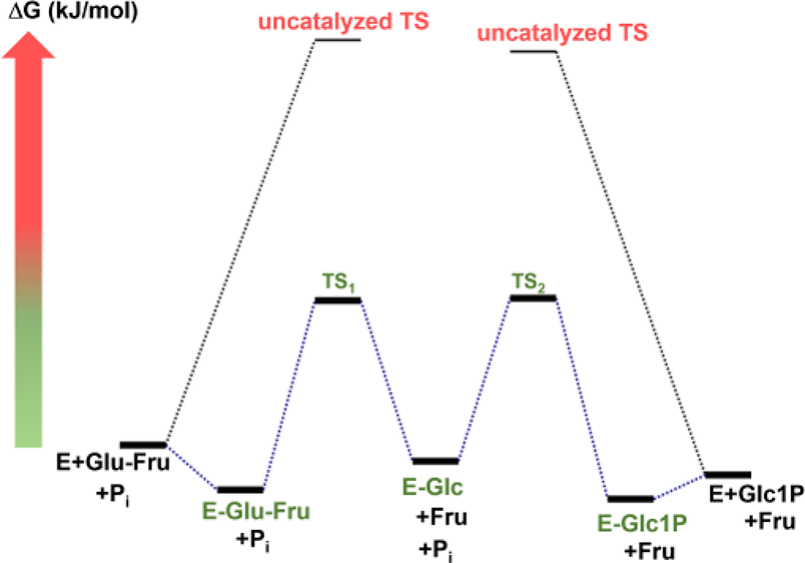

From its structure and mechanism, sucrose phosphorylase is a specialized glycoside hydrolase that uses phosphate ions instead of water as the nucleophile of the reaction. Unlike the hydrolysis reaction, the phosphate reaction is readily reversible and, here, this has enabled the study of temperature effects on kinetic parameters to map the energetic profile of the complete catalytic process via a covalent glycosyl enzyme intermediate. Enzyme glycosylation from sucrose and α-glucose 1-phosphate (Glc1P) is rate-limiting in the forward (*k*_cat_ = 84 s^–1^) and reverse direction (*k*_cat_ = 22 s^–1^) of reaction at 30 °C. Enzyme–substrate association is driven by entropy (*T*Δ*S*_b_ ≥ +23 kJ/mol), likely arising from enzyme desolvation at the binding site for the leaving group. Approach from the ES complex to the transition state involves uptake of heat (Δ*H*^⧧^ = 72 ± 5.2 kJ/mol) with little further change in entropy. The free energy barrier for the enzyme-catalyzed glycoside bond cleavage in the substrate is much lower than that for the non-enzymatic reaction (*k*_non_), ΔΔ*G*^⧧^ = Δ*G*_non_^⧧^ – Δ*G*_enzyme_^⧧^ = +72 kJ/mol; sucrose. This ΔΔ*G*^⧧^, which also describes the virtual binding affinity of the enzyme for the activated substrate in the transition state (∼10^14^ M^–1^), is almost entirely enthalpic in origin. The enzymatic rate acceleration (*k*_cat_/*k*_non_) is ∼10^12^-fold and similar for reactions of sucrose and Glc1P. The 10^3^-fold lower reactivity (*k*_cat_/*K*_m_) of glycerol than fructose in enzyme deglycosylation reflects major losses in the activation entropy, suggesting a role of nucleophile/leaving group recognition by the enzyme in inducing the active-site preorganization required for optimum transition state stabilization by enthalpic forces.

## Introduction

Glycoside hydrolases (GHs) promote the cleavage of glycosidic bonds with rate accelerations of up to ∼10^17^-fold.^1−3^ GHs have drawn mechanistic interest to elucidate the interactions that lead to the impressive proficiency in catalysis.^4−10^ GHs have additionally been studied for the importance and application potential that their transformations have in diverse fields of chemistry, biology, and medicine.^11−17^ Glycoside phosphorylases (GPs) are a specialized group of enzymes within the large GH class.^17−22^ Instead of water as in hydrolysis, the GPs use phosphate as nucleophile of the reaction. Glycosyl transfer to phosphate is called phosphorolysis. Contrary to hydrolysis that involves equilibrium far on the product side, phosphorolysis is readily reversible.^23−26^ The feature of reversibility opens interesting opportunities for synthesis. Various di- and oligosaccharides,^27−30^ and even polymeric glycans,^27,31−33^ have been prepared from glycosyl phosphate substrates using phosphorylase. This has also inspired research into the discovery^34,35^ and engineering^36−39^ of GHs that can use phosphate as the nucleophile.

**Figure 1:**
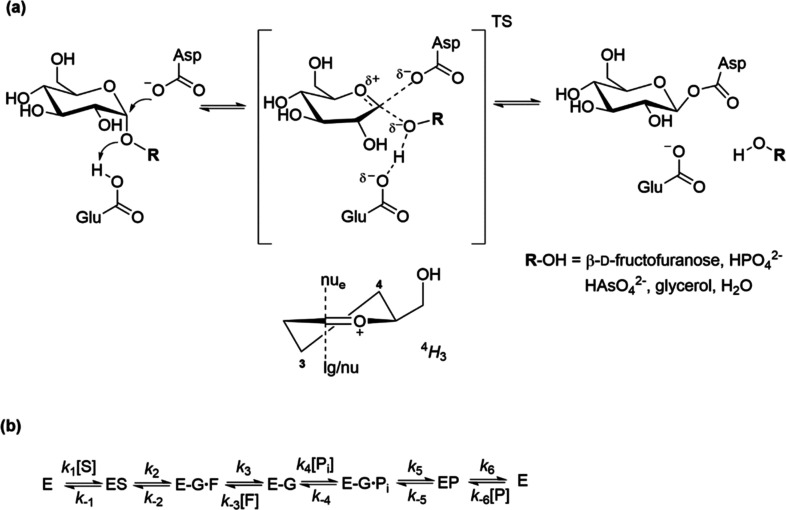
Proposed reaction mechanism of sucrose phosphorylase. (a) Covalent catalysis involving a β-glucosyl enzyme intermediate and proceeding through an oxocarbenium-like transition state (TS). A pair of carboxylic acid residues (Asp: nucleophile, nu_e_; Glu: general acid-base) promote the reaction in two catalytic steps. lg, substrate leaving group; nu, acceptor nucleophile. **R**-OH is shown as lg/nu. (b) Kinetic mechanism of phosphorolysis of sucrose (S) via the glucosyl enzyme intermediate (E–G) after release of fructose (F). Reaction of E–G with phosphate (Pi) gives the product Glc1P (P). Individual microscopic rate constants of the reversible reaction are shown.

**Figure 2:**
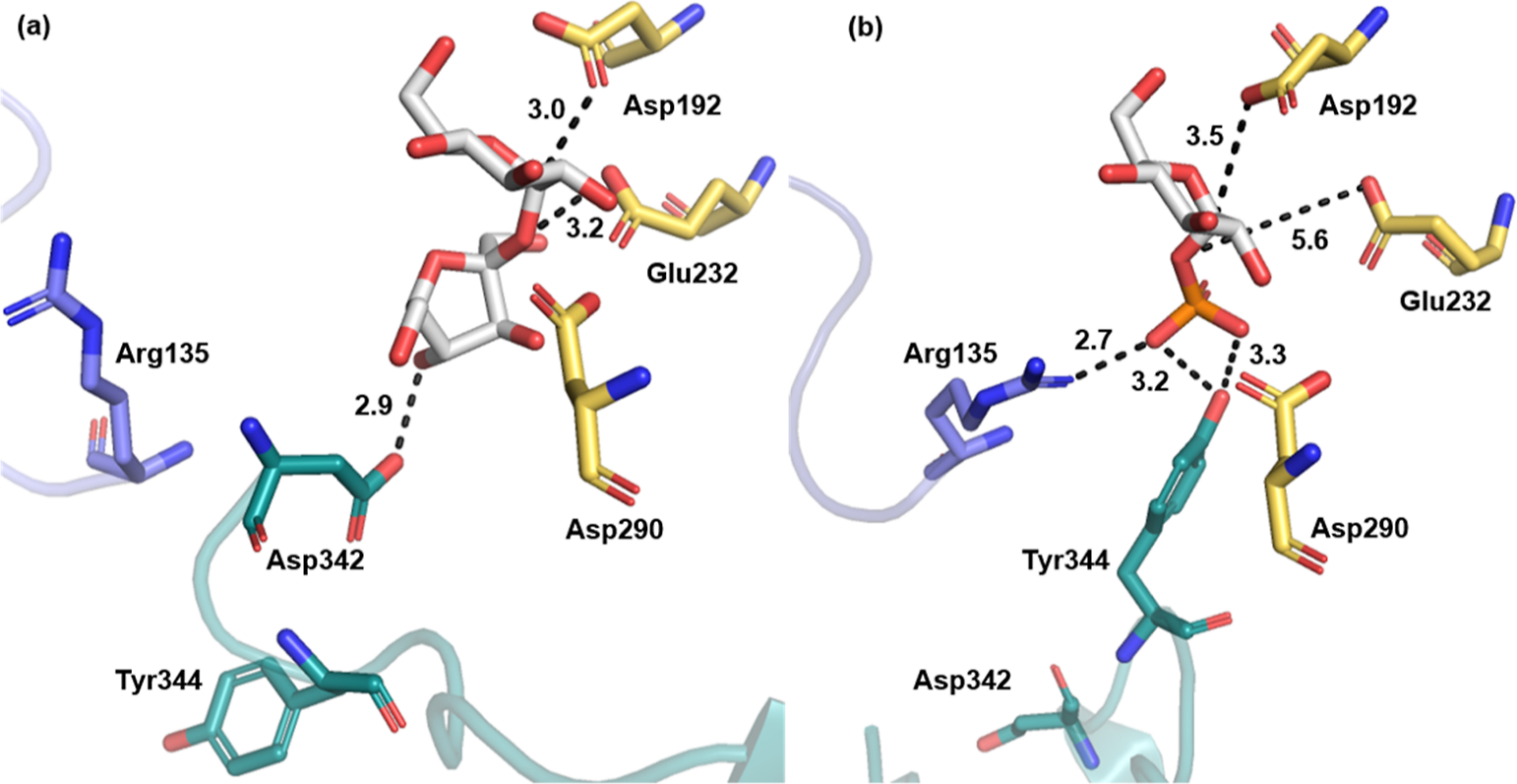
Close-up views of the active-site conformations of *Bifidobacterium adolescentis*(42,44) sucrose phosphorylase bound with (a) sucrose and (b) Glc1P. The model in (a) was obtained from the experimental sucrose-complex structure of a E232Q variant of the enzyme (PDB: 2gdu(44)), with Glu232 substituted in silico. The model in (b) was obtained by docking Glc1P into the experimental structure of the wild-type enzyme bound with glucose (hydrolysis product; PDB: 2gdv(42)).

Like GHs,^2,7^ phosphorylases are distinguished according to whether their catalytic reaction involves a discrete, typically covalent intermediate.^24,40^ Sucrose phosphorylase uses such a type of covalent catalysis (Figures 1; S1(41−43)). Its natural reaction is conversion of sucrose (α-d-glucopyranosyl-(1 → 2)-α-d-fructofuranoside) and phosphate into α-d-glucose 1-phosphate (Glc1P) and fructose. As shown in Figure 1, the catalytic process involves two half-reactions leading to (*glycosylation*) and from (*deglycosylation*) a β-glucosyl-enzyme intermediate.^41−43^ Enabled by conformational flexibility of the enzyme-binding pocket (Figures 2; S1(42,44)), sucrose phosphorylase is permissive regarding the structure of the nucleophile/leaving group used in the reaction.^41,43,45^ Water is also used, however, at a low activity (≤10% of the nucleophile activity in deglycosylation). Broad specificity for the nucleophile and low hydrolysis represent a combination of properties that is rare among GHs and makes sucrose phosphorylase a promising transglycosylase candidate, for synthetic applications.^41^ The enzyme reaction with glycerol is used industrially to produce the cosmetic ingredient 2-O-α-d-glucosyl glycerol from sucrose (Figure S2^46,47^). Reactions with l-ascorbic acid^48^ and glucose^49,50^ provide other commercially relevant products, namely, l-ascorbic acid 2-α-d-glucoside and kojibiose, respectively (Figure S2).

The current study was performed to obtain a better understanding of the energetics underlying the versatile catalysis of sucrose phosphorylase. The approach used was a detailed study of temperature effects on steady-state rate parameters for the main types of enzymatic reaction:^40,47^ phosphorolysis, transglycosylation (to glycerol), and hydrolysis (Figure 1a).

A judicious choice of the temperature range avoided well-known hazards of temperature-rate profile analysis in the mechanistic investigation of enzymes.^51−53^ The evidence obtained reveals thermodynamic changes that accompany substrate binding in the ground state and transition state on both flanks of the covalent intermediate. In both directions of the enzymatic reaction, glycoside bond cleavage in the donor substrate (sucrose, Glc1P) is rate-determining. From comparison with the relevant non-enzymatic reactions (i.e., acid-catalyzed hydrolyses of sucrose^54,55^ and Glc1P^56^) known from the literature to involve glycosidic bond cleavage as the rate-determining step and to proceed through similar oxocarbenium ion-like transition states^57−61^ as proposed for the enzymatic half-reactions (Figure 1a^2,7^), we obtained estimates for the catalytic rate enhancement (∼10^12^-fold) and the transition state affinity (∼10^14^ M^–1^) of sucrose phosphorylase. The forces used in transition state stabilization are primarily enthalpic in origin. Entropy contributes mostly to substrate binding and appears to involve effects of desolvation at the binding site for the nucleophile/leaving group. Insights into the energetics of the phosphorylase reaction(s) can have relevance for GH catalysis in general and may be of interest in ongoing efforts to replace water by phosphate as nucleophile of enzymatic glycoside conversions.^36−39^ Among the GHs employing covalent catalysis, the *Agrobacterium* sp. β-glycosidase^2,4^ and the *E. coli* LacZ β-galactosidase^23,62,63^ have been particularly well studied mechanistically. Like sucrose phosphorylase, the two glycosidases strongly reduce the heat of activation in glycoside bond cleavage reactions catalyzed.^2,23^

## Materials and Methods

### Materials

The chemicals used were of highest reagent quality available from Carl Roth or Sigma-Aldrich. Purified preparation of the *Bifidobacterium longum* sucrose phosphorylase (GeneBank: AAO84039.1; Uniprot: Q8G6U7)^48^ was used (Supporting Information, Methods S1.1 and 1.2). Using standard coupled-enzyme assay (Supporting Information, Methods S1.3.1), the enzyme had a specific activity of 87 U/mg, consistent with earlier work.^48^

### Initial Reaction Rates Determined at Variable Temperature

#### General Procedure

Incubations were made in 1.0 mL liquid volume in 1.5 mL Protein LoBind Tubes placed in a ThermoMixer C (both from Eppendorf), with agitation at 1000 rpm. The temperature range was 5–60 °C. Incubations below room temperature were performed in a cooling room (4 °C). In each reaction, the temperature was controlled at ± 0.5 °C. Substrate solution in 50 mM MES buffer (pH 7.0) was equilibrated to temperature for 30 min. The pH used was verified before and after the reaction. Reaction was started with 10 μL enzyme solution. The protein concentration in the reaction was between 0.3 μg/mL (*T* ≤ 20 °C) and 5.0 μg/mL (*T* ≥ 23 °C). Samples (100 μL) were taken at suitable times, with total reaction time varying between 6 min (*T* ≥ 23 °C) and 30 min (*T* ≤ 20 °C). The sample was heated to ∼100 °C by dilution (≥2-fold), which leads to an instantaneous inactivation of the enzyme.^64^ The sample was analyzed for the product released, as described below. Three or more samples were taken to obtain a time course. Initial rates were calculated from the linear product release with time. The substrate consumption was ≤25% of the maximum conversion in the respective reaction under the conditions used, while considering the effect of the reaction equilibrium.

#### Reactions Analyzed

The reactions analyzed were as follows: phosphorolysis and synthesis of sucrose (Figure 1b); hydrolysis of sucrose in the absence of phosphate; hydrolysis of Glc1P in the absence of fructose; and reaction of sucrose where arsenate replaces phosphate as well as reaction of Glc1P in the presence of arsenate, referred to as arsenolysis of sucrose and Glc1P, respectively. Reported spectrophotometric assays (described in full in the Supporting Information, Methods S1.3) were used to quantitate the product released. In phosphorolysis of sucrose, Glc1P was measured (Supporting Information Methods S1.3.1). Glucose was additionally measured (Supporting Information Methods S1.3.3) but found to be below detection, thus ruling out hydrolysis as a side reaction of phosphorolysis under the conditions used. In synthesis of sucrose, phosphate released from Glc1P was measured (Supporting Information Methods S1.3.2). In hydrolysis of sucrose or Glc1P, glucose was measured (Supporting Information Methods S1.3.3). In arsenolysis of sucrose and Glc1P, α-glucose 1-arsenate was released. Since α-glucose 1-arsenate is not stable, the measured product was glucose. Reaction of sucrose in the presence of glycerol involves transglycosylation to give α-glucosyl glycerol as the product.^46^ Hydrolysis happens at the same time. Fructose and glucose were measured enzymatically (Supporting Information Methods S1.3.3). The transglycosylation rate was calculated as the difference of Δ[fructose]/Δ*t* (= the total reaction rate) and Δ[glucose]/Δ*t* (= the hydrolysis rate).

#### Kinetic Parameters and Their Temperature Dependencies

The catalytic constant (*k*_cat_) and the catalytic efficiency (*k*_cat_/*K*_m_) were determined. Using the general procedure described above, initial rates were recorded at the variable concentration of one substrate while keeping the other substrate concentration constant and saturating. A detailed summary of the experimental conditions used is given in Table S1 (Supporting Information). Although full Michaelis–Menten curves were also recorded, we found it efficient to obtain the *k*_cat_/*K*_m_ from the part of the curve where the rate was linearly dependent on the substrate concentration (see Figure S4 in the Supporting Information for examples). The *k*_cat_ was from conditions of complete saturation of enzyme with substrate. The calculation of *k*_cat_ and *k*_cat_/*K*_m_ was based on the molecular mass of the enzyme subunit (57700 Da) and the protein concentration measured with Bradford assay. Initial rates were recorded in three or more replicates. Averaged rates were used to determine kinetic parameters. Fitted values have standard deviation of 10% or less. Kinetic parameters were obtained in a suitable temperature range, as shown in the Results section.

#### Data Analysis

Temperature profiles were fitted with the Arrhenius (eq 1) and the Eyring equation (eq 2) using linear least-squares regression analysis with SigmaPlot 10.0.
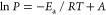
1
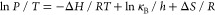
2*P* (kinetic parameter) is *k*_cat_ or *k*_cat_/*K*_m_. In eq 1, *A* is the pre-factor and *E*_a_ is the activation energy. In eq 2, Δ*H* is enthalpy and Δ*S* is entropy. *R* is the gas constant (8.314 J/mol K), κ_B_ is the Boltzmann constant (1.3806 × 10^–23^ m^2^ kg/s^2^ K), *h* is the Planck constant (6.6260 × 10^–34^ m^2^ kg/s), and *T* is absolute temperature (K). The quality of the linear fit was assessed visually, from the coefficient of determination *R*^2^ (≥0.95) and reasonable normal distribution of the residuals. Profiles typically developed curvature at high temperatures. The temperature range used for linear fit was selected according to the stated criteria.

Curved temperature profiles were also fitted globally with a model (eq 3) that assumes both Δ*H* and Δ*S* to be temperature-dependent.^65^ Here, *T*_0_ is the optimum temperature of the enzymatic reaction. The parameter Δ*C*_p_ is the difference in heat capacity between the relevant enzyme states reflected in the kinetic parameter used, as discussed later.
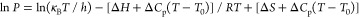
3

#### Free Energy Profile

The free energy profile of the enzymatic phosphorolysis of sucrose (*T* = 303 K) was constructed with the assumption of a 1.0 M standard state. Free energy differences associated with the kinetic parameters were calculated using eqs 4–8. Here, free energies of enzyme–substrate complex (Δ*G*^ES^), transition state (Δ*G*^TS^), equilibrium (Δ*G*^eq^), and covalent intermediate (Δ*G*^int^) were evaluated. *K*_eq_ is the equilibrium constant, while *k*_cat_/*K*_m_ is denoted as *Q* along with its associated substrate mentioned in the subscript.
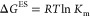
4
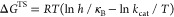
5
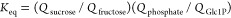
6
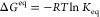
7
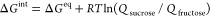
8

#### Enzyme Stability

Experiments were performed to determine whether the enzyme had lost activity during the incubations for initial rate measurement. A sample (10 μL) was taken at the end of each incubation and immediately diluted (33-fold) into the standard coupled-enzyme assay for the phosphorylase activity (Supporting Information Methods, S1.3.1). Only incubations showing the same activity (±5% error) before and after the reaction were considered for detailed analysis of the temperature effect on kinetic parameter.

## Results

### Temperature Profiles for Reversible Phosphorolysis of Sucrose

**Figure 3:**
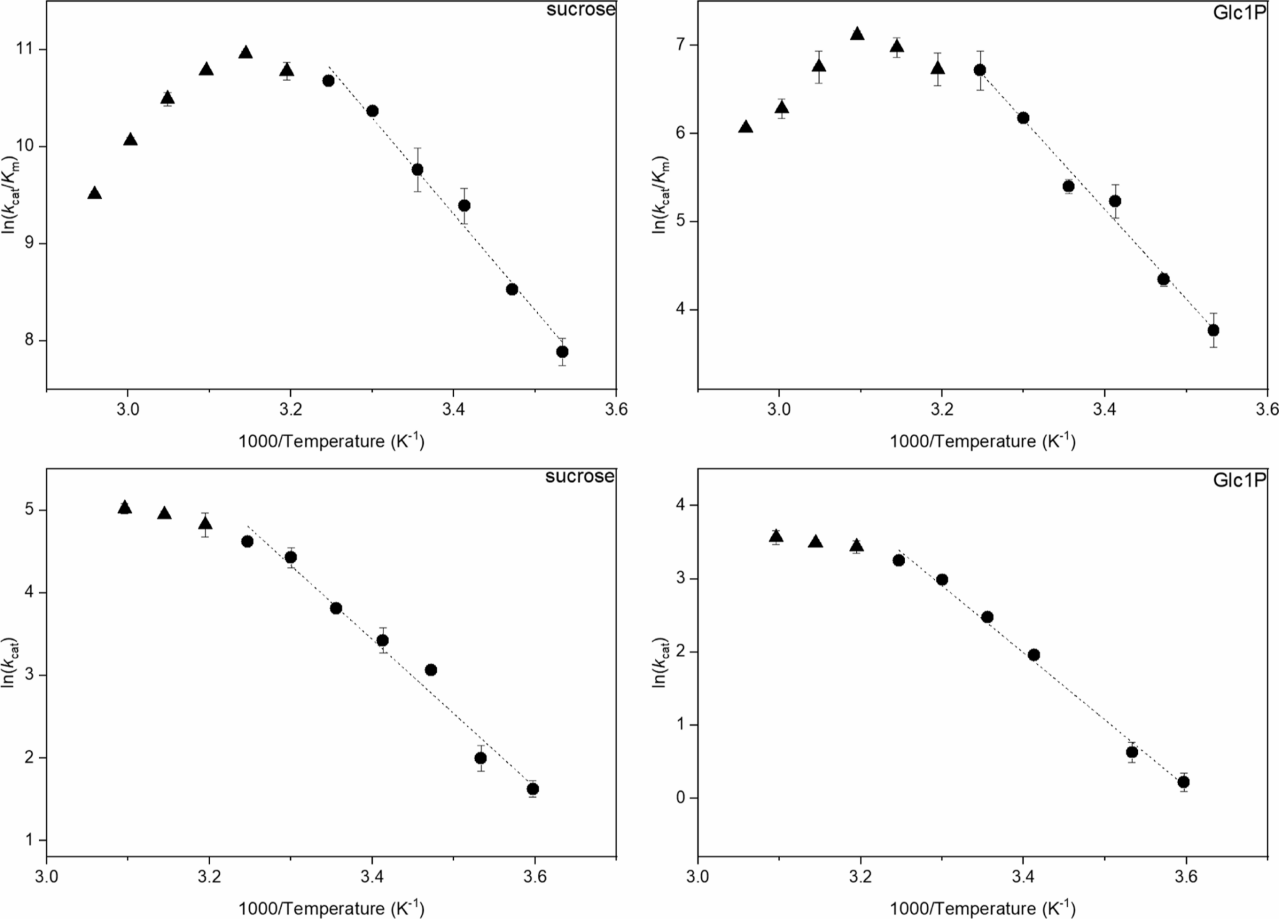
Arrhenius plots for *k*_cat_/*K*_m_ (donor substrate) and *k*_cat_ in both directions of the enzymatic reaction. The circles denote linearly fitted points, while triangles show data not included in the fit. The linearly fitted range (subset of overall data set) is denoted with a dashed line with correlation coefficients ≥0.97.

The *k*_cat_/*K*_m_ for the donor substrate (sucrose, Glc1P) and the corresponding *k*_cat_ in phosphorolysis and synthesis of sucrose were measured. Temperature profiles of the kinetic parameters (Figure 3) were obtained in the range 5–60 °C. The *k*_cat_/*K*_m_ (sucrose) profile showed a maximum at 45 °C, while that of *k*_cat_/*K*_m_ (Glc1P) increased to 50 °C (Figure 3). Enzymes used at ≥0.3 μg/mL and supplemented with BSA (0.5 mg/mL) resisted irreversible inactivation in the ∼6 min of reaction up to 50 °C. At 60 °C, however, ∼20% of the original activity was lost irreversibly in that time. The temperature profiles of the *k*_cat_ for phosphorolysis and synthesis exhibited maximum at ∼50 °C (Figure 3). Like the *k*_cat_/*K*_m_, the *k*_cat_ decreased strongly between 50 and 60 °C. In each case, the percent loss in activity relative to the maximum was much larger (≥10-fold) than expected from the corresponding irreversible inactivation.

### Free Energy Profile of the Phosphorylase Reaction

**Figure 4:**
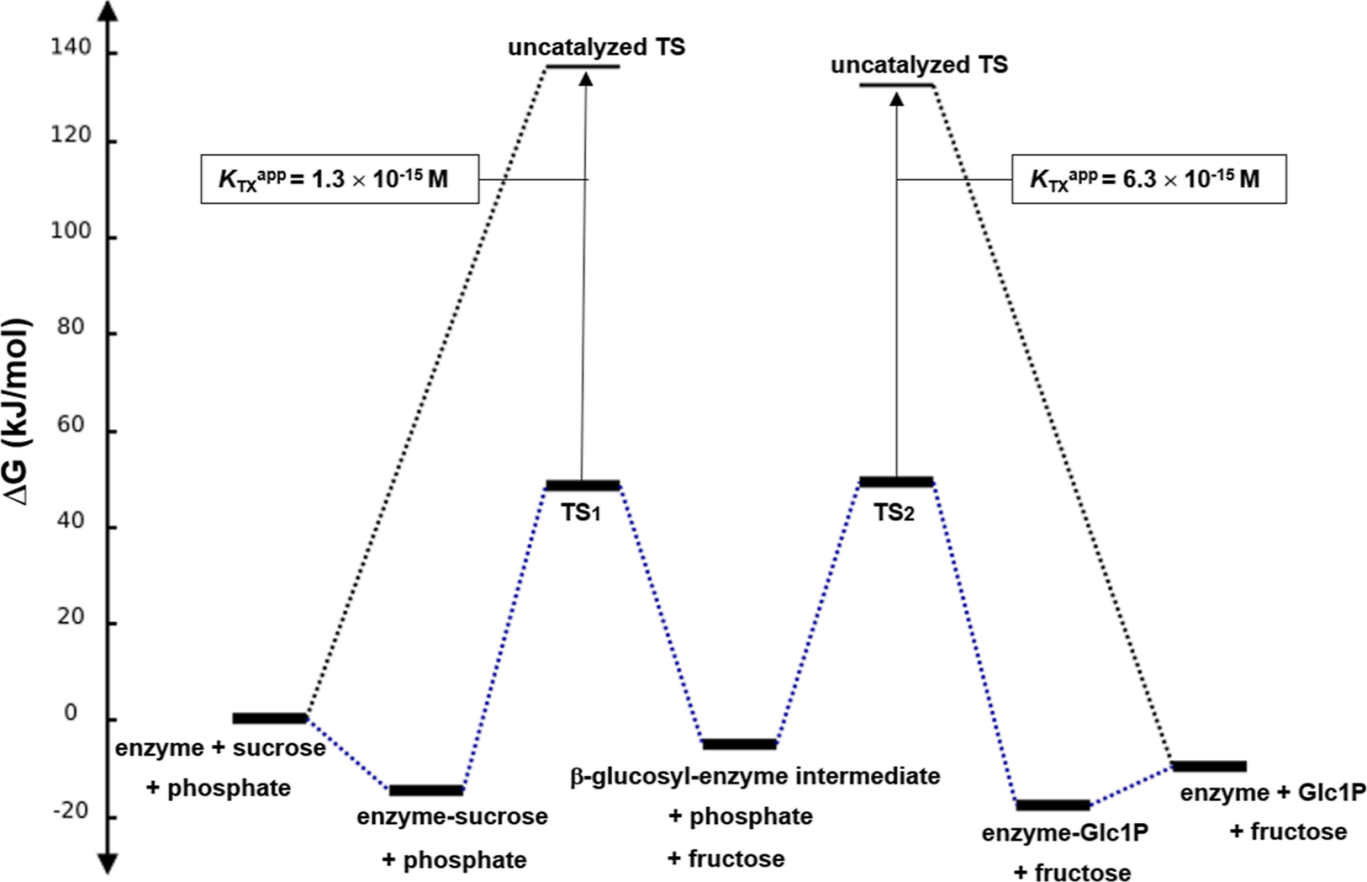
Free energy diagram for the sucrose phosphorylase reaction with sucrose and phosphate. A standard state of 1.0 M was assumed. The temperature was 30 °C, and the pH was 7.0. The diagram was constructed with eqs 4−8 and kinetic parameters from Table 1. Table S2 (Supporting Information) summarizes the free energies shown here graphically. Activation free energies for the non-enzymatic acid-catalyzed hydrolysis of sucrose and Glc1P are added for comparison. First-order rate constants for the non-enzymatic reactions are from Table 1, and eq 5 was used to calculate the free energies of activation. *K*_TX_^app^ describes the binding affinity of the enzyme for the activated substrate in the transition state, as discussed in the Discussion.

Using the *k*_cat_/*K*_m_ values for phosphorolysis and synthesis (Table 1), a basic free energy profile of the phosphorylase reaction at 30 °C was constructed (Figure 4). The internal consistency of the used kinetic parameters was validated with the Haldane relationship (eq 6) that relates the *k*_cat_/*K*_m_ values for enzyme glycosylation and deglycosylation in each direction of the reaction to the equilibrium constant (*K*_eq_).^66^ The *K*_eq_ value of 51 calculated with eq 6 agrees with the *K*_eq_ value of 44 (±60%) determined previously^66^ by experiment.

**Table 1:** Compiled Thermodynamic Parameters of Different Reactions of Sucrose Phosphorylase

reaction	*k*_cat_^a^ (s^–1^), *k*_cat_/*K*_m_^a^ (M^–1^s^–1^), *K*_m_^a^ (M)	kinetic parameter expressed in rate constants from Figure 1b	Δ*H*^⧧^; *E*_a_ (kJ mol^–1^)	Δ*S*^⧧^ (J K^–1^mol^–1^)	Δ*G*^⧧^^a^, Δ*G*_b_^b^ (kJ mol^–1^)
**Phosphorolysis**					
*k*_cat_/*K*_m_ (sucrose)	(32 ± 2.1) × 10^3^	*k*_1_*k*_2_/(*k*_–1_ + *k*_2_)	80 ± 5.1; 82	+104 ± 28	48
*k*_cat_/*K*_m_ (phosphate)	(4.1 ± 0.2) × 10^3^	*k*_4_*k*_5_/(*k*_–4_ + *k*_5_)	86 ± 8.8; 89	+108 ± 30	54
*k*_cat_	84 ± 10	*k*_2_*k*_5_/(*k*_2_ + *k*_5_) *k*_2_*k*_5_/(*k*_2_ + *k*_5_)	72 ± 5.2; 75	+29 ± 18	63
		∼*k*_2_ (≤30 °C)			
1/*K*_m_ (sucrose)^b^	385^c^	*k*_1_(*k*_2_ + *k*_5_)/[*k*_5_(*k*_–1_ + *k*_2_)]	8.0; 7.0	+75	–15
		∼*k*_1_/*k*_–1_ (≤30 °C)			
**Synthesis**					
*k*_cat_/*K*_m_ (Glc1P)	(0.48 ± 0.02) × 10^3^	*k*_–6_*k*_–5_/(*k*_6_ + *k*_–5_)	82 ± 5.2; 85	+77 ± 18	59
*k*_cat_/*K*_m_ (fructose)	(5.4 ± 0.3) × 10^3^	*k*_–3_*k*_–2_/(*k*_3_ + *k*_–2_)	85 ± 9.4; 87	+106 ± 32	52
*k*_cat_	22 ± 2.6	*k*_–5_*k*_–2_/(*k*_–5_ + *k*_–2_)	74 ± 3.5; 76	+23 ± 12	67
		∼*k*_–5_ (≤30 °C)			
1/*K*_m_ (Glc1P)^b^	35^c^	*k*_–6_(*k*_–5_ + *k_−_*_2_)/[*k*_–2_(*k*_6_ + *k*_–5_)]	8.0; 9.0	+54	–8.0
		*∼k*_–6_/*k*_6_ (≤30 °C)			
**Arsenolysis**					
*k*_cat_ (sucrose)	74 ± 11	∼*k*_2_ (≤30°C)	66 ± 3.4; 69	+10 ± 11	63
*k*_cat_ (Glc1P)	27 ± 3.0	∼*k*_*–*5_ (≤30°C)	72 ± 9.4; 74	+24 ± 33	65
**Hydrolysis**					
*k*_cat_ (sucrose)	0.67 ± 0.07	*k*_5_^d^	68 ± 6.0; 71	–22 ± 19	75
*k*_cat_ (Glc1P)	0.75 ± 0.12	*k*_5_^d^	71 ± 6.0; 73	–14 ± 19	75
**Transfer to Glycerol**					
*k*_cat_/*K*_m_, *k*_cat_^e^(glycerol)^e^	4.9 ± 0.3, 9.7 ± 0.6	∼*k*_5_^f^	44 ± 3.8; 47	–80 ± 13	68
**Acid Hydrolysis**					
*k*_chem_ (sucrose)^g^	4.0 × 10^–11^		n.a.; 99 ± 2.0		135
*k*_chem_ (Glc1P)^g^	3.0 × 10^–12^		n.a.; 126		141

a Evaluated at 30 °C.

b Δ*H*_b_, Δ*S*_b_, and Δ*G*_b_ (binding) were calculated as the difference between the parameters (Δ*H*, Δ*S*, and Δ*G*) evaluated from *k*_cat_/*K*_m_ (sucrose or Glc1P) and *k*_cat_ (phosphorolysis or synthesis), respectively.

c The values were obtained from Figure S4 (Supporting Information).

d Enzyme deglycosylation by water.

e Apparent value at 2.0 M glycerol.

f Enzyme deglycosylation by glycerol.

g Values were calculated from literature on the acid-catalyzed hydrolysis of sucrose^55^ and Glc1P,^56^ as shown in the Supporting Information in sections S1.4 and S1.5; n.a., not applicable.

The free energy profile constructed from the *k*_cat_/*K*_m_ values lacks important information on the free energies of binary enzyme–substrate complexes formed in the reaction. Such information might be added from the set of kinetic parameters in Table 1 if the *k*_cat_ was limited by a single microscopic step. Table 1 shows expressions of the kinetic parameters by the microscopic rate constants of the kinetic mechanism in Figure 1b. Linear Arrhenius plots in the low temperature range (≤35 °C; Figure 3) indeed support the suggestion that the *k*_cat_ in both directions of reaction was controlled by one single rate constant. The respective rate-limiting step was localized with data for the arsenolysis of sucrose and Glc1P as follows. Results (*k*_cat_) for the arsenolysis reactions are shown in Table 1. The *k*_cat_ for synthesis (22 s^–1^) was well comparable to the *k*_cat_ for arsenolysis of Glc1P (27 s^–1^). The rate of enzyme deglycosylation to arsenate cannot be smaller than the *k*_cat_ for arsenolysis of sucrose, which is 74 s^–1^ (Table 1). A rate of ∼22 s^–1^ was, therefore, assigned to the step of enzyme glycosylation from Glc1P (*k*_–5_).

The *k*_cat_ for sucrose conversion was unchanged within limits of error when arsenate replaced phosphate as nucleophile of the reaction (Table 1). Thermodynamic parameters of the *k*_cat_ (sucrose) were identical for the phosphate and arsenate reactions in the low temperature range (≤30 °C; Table 1). Despite their overall similarity in chemical structure, arsenate and phosphate differ by ∼11% in the single bond length to oxygen (P–O: 1.54 Å; As–O: 1.71 Å^67^). It was implausible, therefore, that the *k*_cat_ values for the sucrose reactions with phosphate and arsenate would be effectively the same, if enzyme deglycosylation (*k*_5_) were rate-limiting. However, the observed constancy of *k*_cat_ in both reactions was fully consistent with the suggestion of enzyme glycosylation from sucrose (*k*_2_) being the rate-determining step. The steps *k*_2_ (enzyme glycosylation from enzyme-sucrose) and *k*_-4_ (enzyme glycosylation from enzyme-Glc1P) were, therefore, included into the free energy profile and the free energies of the binary enzyme–substrate complexes with sucrose and Glc1P are shown in Figure 4.

Figure 4 also includes free energies for the transition states of acid-catalyzed hydrolyses of sucrose and Glc1P. The first-order rate constants for the non-enzymatic reactions were obtained from the literature, as shown in sections S1.4 (sucrose^55^) and S1.5 (Glc1P^56^) of the Supporting Information and explained in more detail in the Discussion. Comparison of enzymatic and non-enzymatic reactions is also made later in the Discussion.

### Temperature Profiles of Transglycosylation from Sucrose

We determined temperature profiles of the apparent *k*_cat_ for the conversion of sucrose in the absence (hydrolysis) and presence of 2.0 mol/L glycerol (transglycosylation) (Figures S7 and S8). The activation energy for the glycerol reaction (*E*_TG_) was ∼1.5-fold smaller than that for hydrolysis (*E*_H_) (Table 1). Given the low *k*_cat_, enzyme deglycosylation must be rate-limiting in hydrolysis. The *k*_cat_ with its associated activation energy was, therefore, independent of the substrate used (sucrose, Glc1P), as shown in Table 1. In the reaction with glycerol (2.0 M) at 30 °C, the apparent *k*_cat_ was enhanced ∼15-fold over the hydrolysis-only *k*_cat_.

## Discussion

### Energetics of Catalysis by Sucrose Phosphorylase

To obtain a clearer understanding of the energetics involved in transition state stabilization by sucrose phosphorylase, we studied the thermodynamic changes that accompany substrate binding in the ground state and transition state of glycosyl transfer to and from the enzyme. Evidence of temperature profile analysis supports the notion that enzyme glycosylation from sucrose and Glc1P is rate-determining in both directions of the reaction at 30 °C. With the assumption of *K*_m_ to represent an overall enzyme–substrate dissociation constant (Table 1), we show that the productive association between enzyme and substrate (1/*K*_m_) is accompanied by a large gain in entropy (*T*Δ*S*_b_ ≈ +23 kJ/mol for sucrose; +16 kJ/mol for Glc1P) and a somewhat smaller uptake of heat (Δ*H*_b_ = +8.0 kJ/mol for sucrose; Δ*H*_b_ = +8.0 kJ/mol for Glc1P). The entropy increase, which drives the overall binding of both substrates, might be explained by extensive desolvation of the sucrose phosphorylase-binding pocket, and the consequent increase in disordering of the solvent water, on formation of the enzyme–substrate complex. The positive Δ*H*_b_ is compatible with a rearrangement of the hydrogen bond network in the enzyme required to position the substrate for catalysis. On proceeding from the enzyme–substrate complex to the transition state, there is little further change in entropy, but heat is taken up in an amount about twice the heat used in enzyme–substrate complex formation. The *k*_cat_/*K*_m_ that represents the overall enzymatic conversion of the free substrate to the transition state, thus, depends strongly on the temperature (more so than the *k*_cat_), and it involves a substantial increase in entropy of activation that almost matches the entropy gain during substrate binding. Interestingly, the thermodynamic changes accompanying the deglycosylation of sucrose phosphorylase by the nucleophile phosphate or fructose are very similar to the ones accompanying enzyme glycosylation from Glc1P and sucrose, respectively. The *k*_cat_/*K*_m_ for deglycosylation represents the productive association of the covalent glucosyl–enzyme intermediate and the free nucleophile to the transition state. The large increase in entropy of activation accompanying the reactions of free fructose and phosphate supports the idea that, given the requirement for enzyme desolvation in the productive encounter with the substrate, the obligatory release of waters is connected to the binding of the leaving group/nucleophile. Tentatively, one might take the positive entropy of activation additionally as an indication that the enzymatic transition state involves only weak preassociation of the sugar C1 atom with the O atom of the incoming nucleophile of the reaction (see Figure 1a). The *k*_cat_/*K*_m_ for the reaction with glycerol, by contrast, involves negative entropy of activation in sizeable amount. Glycerol, thus, differs from fructose and phosphate by lacking the ability to exploit entropic effects of acceptor substrate binding to the formation of the transition state of deglycosylation.

### Comparison of Enzymatic and Non-Enzymatic Reactions

The thermodynamic changes that accompany chemical hydrolyses of sucrose^55^ and Glc1P (note: the doubly protonated, neutral form^56,59^) provide a frame of reference for analyzing the reactions of sucrose phosphorylase (Table 1). Mechanistically, the chemical reactions are thought to be S_N_1 type processes, involving glycosidic bond cleavage to form an oxocarbenium ion species in the rate-limiting step. Both reactions involve protonic assistance (specific acid catalysis) to the C–O bond cleavage. Experimental and computational studies agree that glycosidic bond cleavage in sucrose happens via formation of both glucosyl and fructosyl oxocarbenium ions.^61,68,69^ The density functional theory-computed energy barriers are almost identical for the glucosyl (+101 kJ/mol) and fructosyl (+97 kJ/mol) cleavage paths.^61^ Preference for reaction via the fructosyl path, as suggested by some experimental evidences,^69^ is explained as a thermodynamic effect: an isomerization leads from the glucosyl oxocarbenium ion into the more stable fructosyl oxocarbenium ion (Figure S9^61^). The slightly extended discussion of sucrose hydrolysis is used here to clarify that mechanistic differences of the *overall reactions* notwithstanding, a comparison of enzymatic and non-enzymatic rates of sucrose conversion is meaningful within the current framework. A large body of experimental-computational evidence on enzymes relevantly similar to sucrose phosphorylase (e.g., retaining glycoside hydrolases) supports the idea that the enzymatic transition states also involve substantial oxocarbenium ion character.^2,6−8^ A recent computational study of the β-glucosyl intermediate of sucrose phosphorylase (from *Bifidobacterium adolescentis*) reacting with resveratrol supports an oxocarbenium-like transition state of enzyme deglycosylation.^70^ In this transition state, the sugar adopts a ^4^*H*_3_ half-chair conformation, as expected. The C1–O bond to the enzyme nucleophile (Asp192) is largely broken (distance: ∼2.6 Å) and the O3 of resveratrol shows weak preassociation with the C1 (distance: ∼2.3 Å).^70^

The second-order rate constant for the acid-catalyzed hydrolysis of sucrose (*k*_acid_) at 30 °C is calculated from literature data^55^ as 4.0 × 10^–4^ M^–1^ s^–1^. At pH 7.0, therefore, the hydrolysis rate constant (*k*_chem_ = *k*_acid_ 10^–pH^) is estimated as 4.0 × 10^–11^ s^–1^. The *k*_chem_ used here is consistent with the uncatalyzed rate of sucrose cleavage obtained in a different study at pH 8.1 (5.0 × 10^–11^ s^–1^; 25 °C).^54^ The rate acceleration achieved by the enzyme (*k*_cat_/*k*_chem_), by a factor of 2.1 × 10^12^, thus corresponds to a lowering of the free-energy barrier to glycosidic bond cleavage (ΔΔ*G*^#^) by 72 kJ/mol on formation of the productive enzyme–sucrose complex. This ΔΔ*G*^#^ must be almost exclusively enthalpic in origin, given the small change in entropy accompanying the conversion of the enzyme–substrate complex to the transition state. Comparison of *k*_acid_ to *k*_cat_/*K*_m_ suggests furthermore that sucrose phosphorylase is 8.0 × 10^7^ more proficient than the proton in breaking the substrate’s C–O bond.

Following Wolfenden,^1^ we define an apparent dissociation constant of the enzyme–substrate complex in the transition state (*K*_TX_^app^; M), as shown in eq 9. The use of eq 9 implies an equilibrium treatment of the transition state.^1,71−73^ The emerging dynamic views of the transition state^72,73^ are not included in this approach.
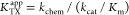
9

The value of *K*_TX_^app^ (= 1.3 × 10^–15^ M), thus, obtained can be compared to the enzyme–substrate dissociation constant in the ground state (∼*K*_m_) of 2.6 × 10^–3^ M, suggesting a 2.0 × 10^12^-fold tighter binding of the substrate in the transition state. We note that the *k*_cat_/*K*_m_ value for sucrose (Table 1) is by far lower than the theoretical limit of a diffusion process (≥10^8^ M^–1^ s^–1^). There can be several reasons for a low *k*_cat_/*K*_m_ in enzymatic reactions. Among these, conformational restrictions of the enzyme in formation of the reactive Michaelis complex seem to be a plausible explanation. Conformational effects might involve additional step(s) of physical binding, e.g., coupled motion^74^ of the initially formed enzyme–sucrose complex to populate the catalytic enzyme conformer that shows electrostatics and internuclear distances tuned for chemical bond cleavage. Alternatively, a relatively rare form of the enzyme might be required for productive encounter. Extensive sampling from the ensemble of enzyme conformers might, thus, cause the *k*_cat_/*K*_m_ to be lowered, yet still represent the activation barrier for substrate binding in the transition state. If physical binding were to limit the *k*_cat_/*K*_m_, the calculated *K*_TX_^app^ would underestimate the true transition state affinity. It is generally difficult, and there is no evidence from this study, to distinguish between the possible mechanisms of conformational control by the enzyme. We, therefore, emphasize the apparent nature of *K*_TX_^app^ as a transition state dissociation constant. Furthermore, we note that the comparison between *K*_TX_^app^ and *K*_m_ remains valid, since both parameters are affected by the physical binding in the same way.

Analysis of the enzymatic reaction with Glc1P has mechanistic interest due to the substantive differences in structure and leaving group ability of phosphate compared to fructose. It is further of interest in light of crystallographic evidence for conformational flexibility (i.e., coupled motions involving the rearrangement of active-site loops) used by sucrose phosphorylase to enable reaction with sucrose and Glc1P as the donor substrate (Figure 2^42,44^). Reliance on general acid catalysis to glycosidic bond cleavage is effectively eliminated in the enzymatic reaction with Glc1P compared to sucrose (Figure S1^75^). Interestingly, therefore, the free energy barriers for the enzymatic reaction (*k*_cat_/*K*_m_) with Glc1P (Δ*G*^#^ = +59 kJ/mol) and sucrose (Δ*G*^#^ = +48 kJ/mol) are similar. The rate acceleration achieved by the enzyme is 6.7 × 10^12^-fold at pH 7.0, relative to hydrolysis of the neutral Glc1P (*k*_chem_ = 3.0 × 10^–12^ s^–1^).^56^ The non-enzymatic rate was extrapolated from the pH dependence of the acid-catalyzed hydrolysis of the neutral Glc1P in the pH range ≤3.5 (Figure S10).^56^ Extrapolation was required because at the pH of the enzymatic reaction (7.0) the Glc1P exists largely as the monoanion and this hydrolyzes via P–O rather than C–O bond cleavage.^59^ We, thus, note the change in reaction mechanism brought about by the enzyme at neutral pH. The calculated rate acceleration corresponds to a lowering of the free-energy barrier to C–O heterolysis of 74 kJ/mol on formation of the enzyme-Glc1P complex. Using eq 9, the *K*_TX_^app^ for Glc1P is calculated as 6.3 × 10^–15^ M reflecting a 4.6 × 10^12^-fold tighter binding of this substrate in the enzymatic transition state compared to the ground state (29 × 10^–3^ M). The results furthermore suggest that the transition state affinity of Glc1P is similar to that of sucrose. Interestingly, sucrose phosphorylase exhibits a *K*_TX_^app^ for sucrose that is comparable to that of invertase (1.2 × 10^–16^ M).^54^

It may be noted that non-enzymatic C–O bond cleavages in sucrose, Glc1P, and simple glycosides in general are accompanied by an increase in the entropy of activation, typically in the range 20–30 kJ/mol at 30 °C.^60^ Interpretation of this positive change in *T*Δ*S*^#^ usually considers the requirement for reorganization of the solvent cage around the reacted species. As the C–O bond is broken, there is dispersal of charge and a consequent disordering of the solvent shell around the incipient oxocarbenium ion. In the enzymatic transition state, the extent of the solvent shell will be considerably reduced and the entropic effect of the reaction in solution mitigated, if not eliminated entirely. The positive change in *T*ΔS^#^ for the enzymatic reaction (*k*_cat_/*K*_m_), larger than the change for the uncatalyzed reaction in solution, is therefore unlikely to arise from an intrinsic barrier to the chemical change at the transition state. The release of enzyme-bound waters on association of the enzyme with the substrate thus provides a plausible picture of the origin of rise in the entropy of activation in the enzymatic compared to the non-enzymatic reaction.

### Different “Temperature Optima” of Sucrose Phosphorylase

The temperature dependence of the kinetic parameters (*k*_cat_, *k*_cat_/*K*_m_) of sucrose phosphorylase differs by up to ∼5–10 °C in the observed *T*_m_ (Figure S11). In no case of the kinetic parameters was the decrease in rate at high temperatures due to an irreversible process of enzyme inactivation. Three effects could, thus, explain the curvature in Arrhenius plots of enzyme activity versus temperature: a change in the rate-limiting step; thermally induced sampling of enzyme conformers that exhibit low activity, if any; and a negative activation heat capacity for the rate-limiting step.^76^ Note that we include temperature-dependent ionization of a critical enzyme residue among the effects of conformational sampling. Recently, the idea inspired from macromolecular rate theory,^65^ that a heat capacity lower in the transition state than in the relevant ground state of the reaction leads directly to the prediction of a temperature optimum for the rate-limiting step, has attracted considerable interest.^76−79^

The *k*_cat_/*K*_m_ for the donor substrate (sucrose, Glc1P) reflects the difference in free energy between the transition state of enzyme glycosylation and the free standard states of enzyme and substrates. The difference of ∼5 °C in the *T*_m_ of *k*_cat_/*K*_m_ for Glc1P and sucrose does not appear to be consistent with effects of conformational sampling. There is no evidence that the substrate is required to stabilize the enzyme, nor that fructose (present at 50 mM in the reaction with Glc1P) is more stabilizing than phosphate (present at 50 mM in the reaction with sucrose). The sampling of less active enzyme conformers at high temperatures may not reasonably be so different under the conditions used to determine *k*_cat_/*K*_m_ for sucrose and Glc1P in order to account for the substantial difference in the observed *T*_m_. Our discussion considers the possibility of a temperature-dependent E ↔ E′ equilibrium,^80^ where E and E′ are the free enzyme conformers reacting with sucrose and Glc1P, respectively. Note that even though reaction of Glc1P does not require protonic assistance in the way sucrose does,^75^ the active-site protonation state of sucrose phosphorylase must be the same in both reactions (Figure 1, Figure S1). Change in the distribution of total enzyme to favor the Glc1P-reactive E′ at high temperatures might explain a higher *T*_m_ of the *k*_cat_/*K*_m_ for Glc1P than sucrose.

Considering the idea of heat capacity change (Δ*C*_p_) along the reaction coordinate, we fitted the *k*_cat_/*K*_m_ temperature profiles with the extended Arrhenius model in eq 3 that involves temperature dependence of both enthalpy and entropy of activation (Figure S12, Table S3; Supporting Information). The Δ*C*_p_ is, thus, the difference in heat capacity between the transition state and the free enzyme state. The estimated Δ*C*_p_ was −4.1 (±0.50) kJ/(mol K) and −3.2 (±0.40) kJ/(mol K) for the reaction with sucrose and Glc1P, respectively (Table S3). The ΔΔ*C*_p_ of 0.9 kJ/(mol K) was considered to be significant.^65^ A heat capacity of the transition state differing for the reaction with sucrose and Glc1P does not seem to be consistent with the suggestion that both reactions involve a similar oxocarbenium ion-like transition state (Figure 1a). Besides the variable Δ*C*_p_, the extended Arrhenius model was implausible because its fit gave a negative value of the estimated Δ*H* (Table S3). The implied heat release (rather than uptake) upon moving from the free enzyme to the transition state was not physically meaningful, and the Δ*C*_p_ model was thus excluded from further consideration.

A parsimonious explanation for *T*_m_ of *k*_cat_/*K*_m_ lower with sucrose than Glc1P is that the sucrose reaction features a change in the rate-determining step upon increase of the temperature.^53^ The activation energy for *k*_cat_/*K*_m_ is a complex quantity (see ref (53) for a detailed discussion). Depending on the individual rate constants and their corresponding activation energies, the temperature profile of the *k*_cat_/*K*_m_ will change and a different *T*_m_ is possible for the same free enzyme without the need to appeal to a catalytically heterogeneous ensemble of protein conformers. Given the similar *E*_a_ values for the catalytic step of enzyme glycosylation from sucrose (*k*_2_) and Glc1P (*k*_–5_), as shown in Table 1, these considerations imply that the activation energy for Glc1P release from the enzyme is lower than that for sucrose release. The higher *T*_m_ of ∼50 °C observed in the temperature dependence of the *k*_cat_/*K*_m_ for Glc1P as well as the *k*_cat_ for phosphorolysis and synthesis probably reflects an equilibrium shift in the conformational landscape of sucrose phosphorylase at high temperature to increasingly populate enzyme conformers impaired in catalysis.^80^

In conclusion, temperature effects on enzymatic rates reveal thermodynamic characteristics of substrate binding and catalysis by sucrose phosphorylase. This enables comparison of the enzymatic with the non-enzymatic reaction to determine rate enhancement and to estimate transition state affinity. The interactions involved in transition state stabilization are largely enthalpy-based, consistent with the highly polar active site of the enzyme. Entropy changes contribute primarily to the substrate binding and appear to involve enzyme desolvation at the binding site for the leaving group/nucleophile. Despite the difference in their chemical structure, fructose and phosphate exhibit virtually identical activation parameters of their reactions. This suggests that both groups are well-positioned in the enzyme for the catalysis to proceed optimally. Reactions of poor nucleophiles such as glycerol and water are characterized by a substantial negative entropy of activation. The ∼10^3^-fold lower reactivity (*k*_cat_/*K*_m_) of glycerol than fructose is entirely due to entropic effects. Unlike fructose, glycerol seems to be unable to induce the active-site preorganization required for an optimized stabilization of the transition state via enthalpic forces. Overall, sucrose phosphorylase is revealed as a highly efficient catalyst of glycoside bond cleavage, achieving rate accelerations of ∼10^12^-fold. Within the mechanistic framework of retaining glycoside hydrolases,^2,7^ catalytic efficiency of the phosphorylase relies on the fine balance between conformational flexibility and precise positioning. Flexibility enables the two chemically different leaving groups/nucleophiles to be accommodated in the enzyme binding pocket. Positioning tunes the active-site electrostatics and the internuclear distances for bond cleavage/formation. Here, our study emphasizes the subtle level of structural and electronic discrimination that a glycoside hydrolase engineered for reactivity with phosphate^36−39^ would have to exhibit in order to rival the proficiency of the natural phosphorylase. Finally, the difference in *T*_m_ of kinetic parameters of the sucrose phosphorylase provides the basis for a discussion of the mechanisms leading to optimum temperature of the enzymatic rates under conditions in which thermal denaturation is still insignificant.^81^

## Supporting Information Available

Expression and purification of sucrose phosphorylase; reaction conditions used in temperature dependent reactions; assays used for measurement of products; equations used for the construction of energy profile; estimation of uncatalyzed hydrolysis rate of sucrose and Glc1P; commercially relevant products synthesized by sucrose phosphorylase; structure of sucrose phosphorylase; plots for conversion rates versus substrate concentration; Arrhenius plots for various reactions assisted by sucrose phosphorylase; formation of fructosyl oxocarbenium ion; extrapolation of literature data to estimate Glc1P acid hydrolysis rate; shift in *T*_m_ for glycosylation reactions by sucrose and Glc1P; and temperature dependence of *K*_m_ (PDF)
Conformational changes of sucrose phosphorylase during binding of sucrose and Glc1P (MPG)

## Supplementary Material

bi3c00080_si_001.pdf

bi3c00080_si_002.mpg

## Notes

Protein/Enzyme used is *Bifidobacterium longum* sucrose phosphorylase (GeneBank: AAO84039.1; Uniprot: Q8G6U7).

## Abbreviations

Glc1P: α-d-glucose 1-phosphate
GH: glycoside hydrolase
GP: glycoside phosphorylase
