# Supplementary material for: Energetics of the Glycosyl Transfer Reactions of Sucrose Phosphorylase
Source: Biochemistry. 2023 May 30;62(12):1953–63. doi: 10.1021/acs.biochem.3c00080 (PMC10286314; doi:10.1021/acs.biochem.3c00080)
Supplement: Supplementary file 1 — bi3c00080_si_001.pdf [file bi3c00080_si_001.pdf]

Supporting Information

Energetics of the Glycosyl Transfer Reactions of Sucrose Phosphorylase

Anisha Vyas[1], Bernd Nidetzky[1,2]*

[1]Institute of Biotechnology and Biochemical Engineering, Graz University of Technology, Petersgasse 12, A-8010 Graz, Austria.

[2]Austrian Centre of Industrial Biotechnology, Krenngasse 37, 8010 Graz, Austria.

*Corresponding author: bernd.nidetzky@tugraz.at

Supporting materials and methods, pages S4 – S7

Supporting figures, pages S8 – S20

Supporting tables, pages S21 – S23

Supporting video, pages S24

Supporting references, pages S25 – S26

Table of contents

S1. Supporting materials and methods……………...……………………………………...……S4

S1.1. Recombinant gene expression……………………………………………...……...S4

S1.2. Protein purification…………………………………………………………...…S4

S1.3. Measurement of analyte…………………………………………………..……….S5

S1.3.1. Glc1P analysis………………………………………………………...…….S5

S1.3.2. Phosphate analysis…………………………………………………...…..S5

S1.3.3. Glucose and fructose analysis……………………………………………...…S6

S1.4. Estimation of acid hydrolysis rate of sucrose…….……………...……………..…....……...S6

S1.5. Estimation of acid hydrolysis rate of Glc1P………………...…………….…....……….S6

S2. Supporting figures……...……………………………………………………………S8

Figure S1. Differential binding of sucrose and Glc1P to the enzyme…………………………...S8

Figure S2. Production of commercially relevant products using sucrose phosphorylase….........S9

Figure S3. Homodimeric structure of sucrose phosphorylase………………………………….S10

Figure S4. Plots for conversion rate versus substrate concentration…..……………………….S11

Figure S5. Arrhenius profiles for deglycosylation of the enzyme….……………....................S12

Figure S6. Arrhenius profiles for arsenolysis reactions...………………………………………S13

Figure S7. Arrhenius profiles for hydrolysis reactions...…………………………………........S14

Figure S8. Arrhenius plot for transglycoslyation by glycerol………………………………….S15

Figure S9. Formation of fructosyl oxocarbenium ion like transition state..……………………S16

Figure S10. Extrapolation of the pH dependent acid hydrolysis rate..………………….............S17

Figure S11. Shift in T_m for glycosylation reactions by sucrose and Glc1P.....…………………S18

Figure S12. Model fitting to determine specific heat capacity…………………………………S19

Figure S13. Arrhenius profiles for temperature dependent K_m data……………………………S20

S3. Supporting tables...…………………………………………………………………S21

Table S1. Conditions used for temperature dependent reactions ……………………………...S21

Table S2. Free energies plotted in the reaction coordinate diagram……………………..........S22

Table S3. Specific heat capacities and associated parameters……………………………........S23

S5. Supporting video…………...……………………………………………………..........S24

Video S1. Loop movements in the active site towards differential binding……………........S24

S6. Supporting references…………...…………………….……………………………........S25

S1. Supporting materials and methods

S1.1. Recombinant gene expression

Escherichia coli BL21-Gold (DE3) cells harboring recombinant plasmid encoding sucrose phosphorylase (reported in literature) [1] were cultivated in baffled shaken flasks (1 L) at 37 °C using 500 mL LB media (5 g L^{-1} NaCl, 5 g L^{-1} yeast extract, 10 g L^{-1} peptone) supplemented with 115 μg L^{-1} ampicillin. The culture flasks were agitated at 110 rpm using an incubator shaker, CERTOMAT BS-1 (Sartorius, Germany). Gene expression was induced by the addition of 250 μM IPTG at OD_{600} 0.8 – 1.0 and the cells were incubated overnight at 25°C. Cells harvested by centrifugation at 6,000 g (4°C) for 20 min were resuspended in 50 mM potassium phosphate buffer, pH 7.0 to a concentration of 100 g L^{-1}. The cells were disrupted by ultrasonication using Sonic Dismembrator Model 505 (Fisher Scientific, USA) at 60% amplitude comprising of three cycles of 6 min (2 s pulse on and 4 s pulse off). Cell debris were removed by ultracentrifugation at 30,000 rcf (4°C) for 45 min. The resulting supernatant was filtered using 0.45 μm cellulose acetate filter (Sartorius, Germany) and was further used for purification.

S1.2. Protein purification

The purification steps were carried out with an Äkta explorer system (GE Healthcare, Germany) at 4°C assisted with protein detection at 280 nm. Volume of 50 ml of cell free extract (50 mg protein/mL) was applied to nickel sepharose high performance affinity resin packed HisTrap column (16 × 25 mm, 5 mL; GE Healthcare, Germany) equilibrated with 20 mM sodium phosphate buffer (pH 7.0) containing 0.5 M sodium chloride and 50 mM imidazole. All buffer solutions used were filtered using 0.45 μm cellulose-acetate filter (prior to use). Gradient elution was achieved at a constant flow rate of 2 mL min^{-1} using 20 mM sodium phosphate buffer pH 7.0, containing 0.5 M sodium chloride and 0.5 M imidazole. The enzyme was eluted at 100 – 150 mM imidazole and

was concentrated by ultrafiltration using Vivaspin Turbo 15 tubes (Sartorius, Germany) with 30 kDa molecular cut off. Buffer solution was changed to 50 mM potassium phosphate buffer (pH 7.0). The presence of purified recombinant sucrose phosphorylase was confirmed by running a sodium dodecyl sulphate-polyacrylamide gel electrophoresis (SDS-PAGE) in a precast 4 – 12 % gradient gel on an Xcell surelock system (both from Invitrogen, Austria). Bands were visualized by staining the gel using Coomassie Brilliant Blue dye based on molecular weights. Aliquots of 250 μL of purified enzyme (50 mg/mL) supplemented with 5 % glycerol were stored at −20°C for further use (temperature dependent reactions and immobilization).

S1.3. Measurement of analyte

Time dependent samples from temperature-controlled reactions were analyzed for product(s) based on the conversion assisted by sucrose phosphorylase (Figure 1a). Procedures to analyze different compounds entailed in this study are described as following.

S1.3.1. Glc1P analysis

α-Glucose 1-phosphate (Glc1P) concentrations were determined using a colorimetric assay where the formation of NADH was detected at 340 nm using 3.1 mM NAD^+, 2.7 U glucose-6-phosphate dehydrogenase (G6PDH) and 3.1 U phosphoglucomutase in 50 mM Tris/HCl buffer (pH 7.7) with 10 mM magnesium chloride and 10 μM α-D-glucose 1,6-bisphosphate. Reagent to sample (or standard) volume was kept as 114 μL to 80 μL. And the samples were analyzed against a standard curve by reading absorbances on a plate reader at 340 nm.

S1.3.2. Phosphate analysis

Phosphate analyses were performed using a colorimetric assay [2]. Here, molybdate reagent consisting of 15 mM ammonium molybdate and 100 mM zinc acetate at pH 5.0 was mixed with 10% (m/v) L-ascorbic acid in a ratio of 4:1 respectively. 20 μL of sample was dispensed to this

reagent (140 μL) and incubated for 15 mins at 30°C. Reduction of phosphomolybdate complex was read at 850 nm in a microplate reader. And the quantification was based on a standard curve.

S1.3.3. Glucose and fructose analysis

The analysis of glucose and fructose was based on a coupled enzymatic assay using hexokinase (HK) and G6PDH [3]. The measurement was performed using a commercial assay kit (K-SURFG, Megazyme, Ireland). The commercial buffer was mixed with $NADP^+$/ATP and HK/G6PDH to prepare the reagent (8:8:1 by volume respectively) [4]. This reagent (140 μL) was added to samples (80 μL) and the end point absorbances were read at 340 nm (after 20 mins) to estimate glucose concentration [4]. After reading the absorbances for glucose in a microplate reader, an additional 20 μL of 40 times diluted phosphoglucose isomerase solution was added to quantify fructose concentration [4]. Quantification of either analytes was based on a standard plot.

S1.4. Estimation of acid hydrolysis rate of sucrose

The uncatalyzed rate of acid hydrolysis of sucrose was estimated from literature [5] where pH dependent acid hydrolysis rate (k_{acid}) is expressed in terms of activation energy (E_a = 99 kJ mol^{-1}) and its associated rate constant (ln k_0 = 35.6) [5]. The relationship was used (Equation 8 described in the paper) [5] for the estimation. The rate of acid hydrolysis of sucrose was calculated as 4×10^{-4} s^{-1}, while at pH 7.0 ($k_{chem} = k_{acid}\, 10^{-pH}$), the rate was estimated as 4×10^{-11} s^{-1}.

S1.5. Estimation of acid hydrolysis rate of Glc1P

The uncatalyzed rate of Glc1P hydrolysis via C1-O bond cleavage was estimated from literature [6]. For comparative evaluation with sucrose phosphorylase catalyzed reactions, it's crucial that uncatalyzed hydrolysis rate is determined at pH 7.0 and 30°C. The pH dependent logarithmic plot of rates (at 82°C) [6] in the range of pH 1.23 – 3.33 (Figure S10) denotes C1-O bond cleavage (in acidic region). To estimate the rate at pH 7.0, extrapolation was performed (Figure S10) and the

rate was calculated at 82°C (5.7×10^{-9} s^{-1}). Following it, the rate at 30°C was estimated using the activation energy (temperature dependence). The calculation was done by equating the slope i.e. $-E_a/R$ ($E_a = 31$ kcal mol^{-1}) [6] to the ratio of rise and run of the Arrhenius equation. The rate obtained from the calculation at pH 7.0 and 30°C is 3.0×10^{-12} s^{-1}.

S2. Supporting figures

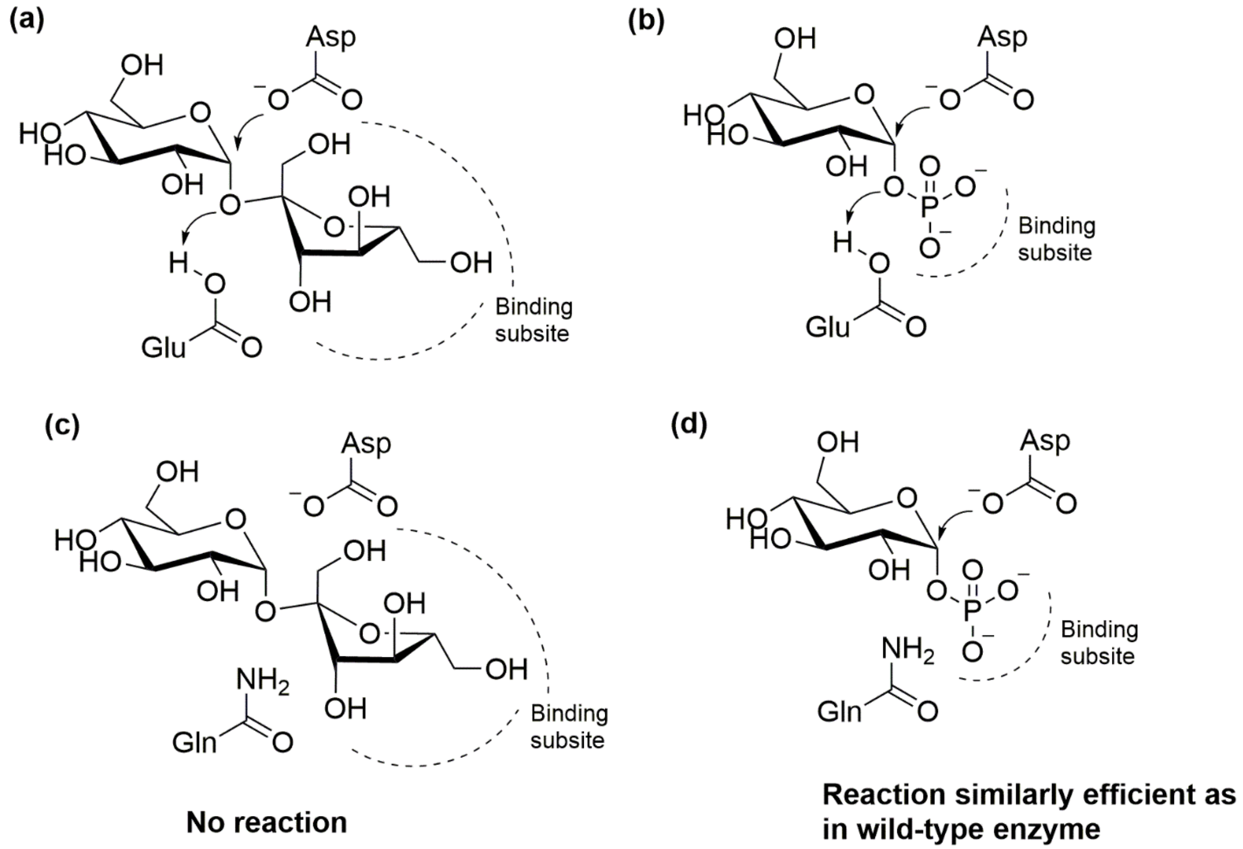

Figure S1. Enzyme glycosylation from sucrose and Glc1P by wild-type and acid-base Glu variant of sucrose phosphorylase. Retention of activity for enzyme glycosylation from Glc1P, but loss of the same for glycosylation from sucrose, was shown for the relevant Glu-to-Gln variant of the sucrose phosphorylase from *Leuconostoc mesenteroides* [7]. The catalytic Glu is highly conserved in sucrose phosphorylase, including the enzyme from *Bifidobacterium longum* used here [1]. To accommodate D-fructosyl or phosphate residue at the subsite for the leaving group (subsite –1), the enzyme needs to undergo conformational change (see Figure 2 of main text).

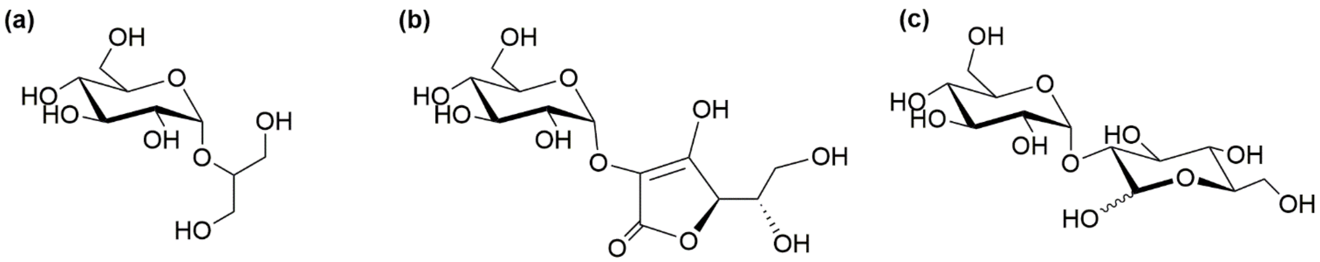

2-O-D-glucosyl glycerol **L-ascorbic acid 2-α-D-glucoside** **kojibiose**

Figure S2. Commercially relevant products synthesized by transglycosylation from sucrose to different glucosyl acceptors such as (a) glycerol, (b) L-ascorbic acid and (c) D-glucose assisted by sucrose phosphorylase.

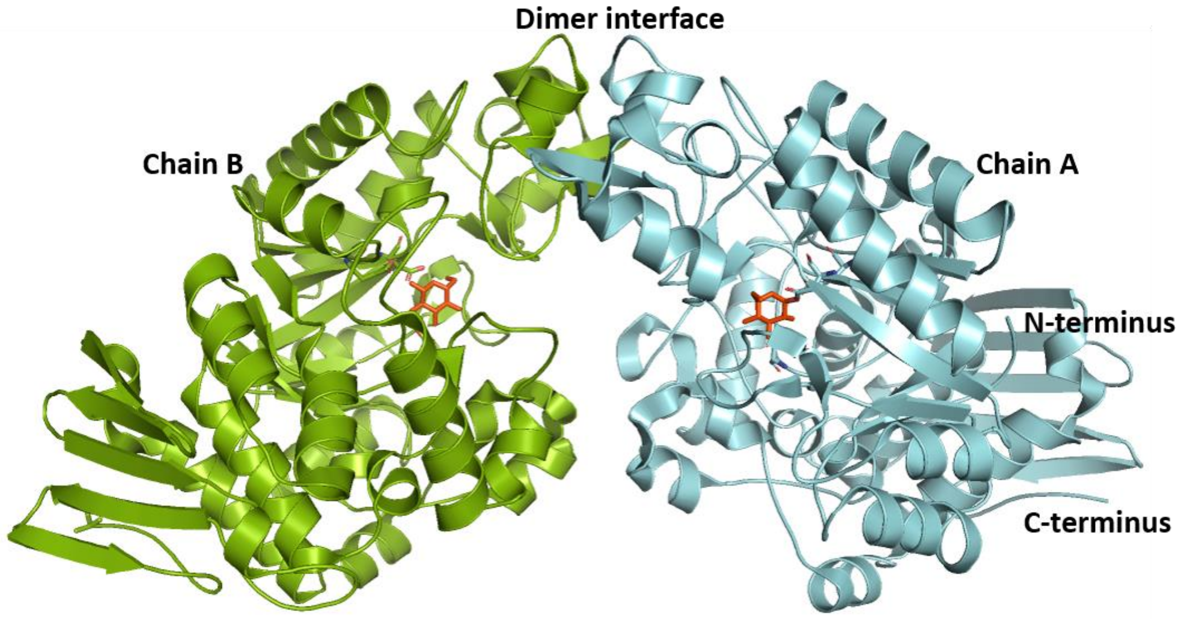

Figure S3. Homodimeric structure of sucrose phosphorylase from *Bifidobacterium adolescentis* (PDB: 2gdv) with chain A (cyan) and B (green) possessing 92 % sequence identity to sucrose phosphorylase from *Bifidobacterium longum*. Alignment of the protein with the alpha fold generated model of the latter yields in RMSD of 0.3 Å depicting structural similarity of the two. The structure shown is reacted with sucrose via the formation of *β*-glucosyl-enzyme intermediate indicated by glucosyl moieties (orange) in the catalytic active site.

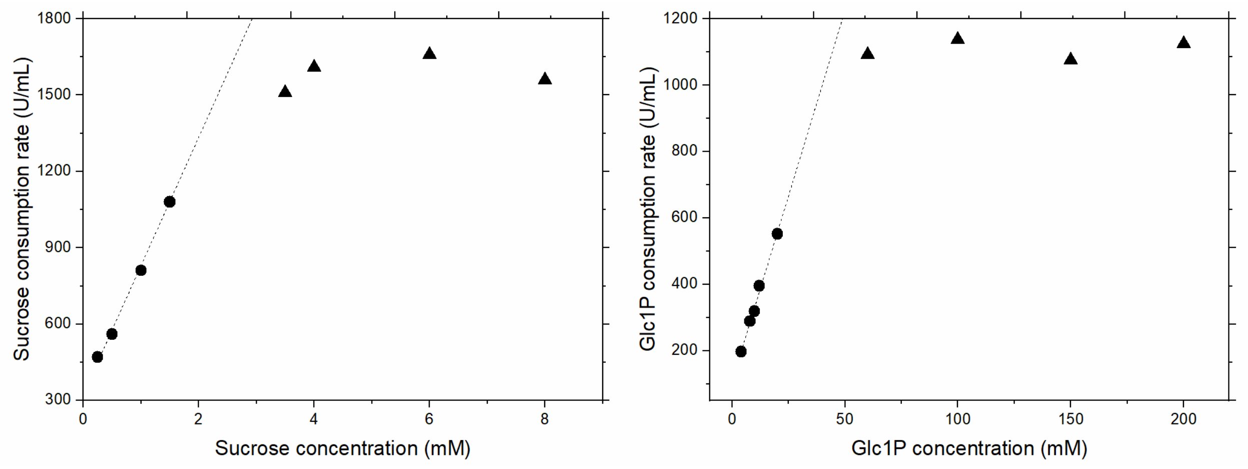

Figure S4. Plots for conversion rates versus substrate concentrations for phosphorolysis (left) and synthesis (right) reactions assisted by sucrose phosphorylase, where sucrose and Glc1P concentrations were varied respectively at 30°C (pH 7.0). The concentration of the second substrate (50 mM phosphate, 50 mM fructose) was constant and saturating. Experimental data are represented as circles and the linear region in the plot is highlighted with a dashed line. The correlation coefficient of the fits here is 0.99. Enzyme added to the phosphorolysis and synthesis reactions were 0.5 and 3 μg per 1 mL reaction respectively. The plots are an exemplification towards determination of a range of substrate concentration where rates vary linearly to determine k_{cat}/K_m.

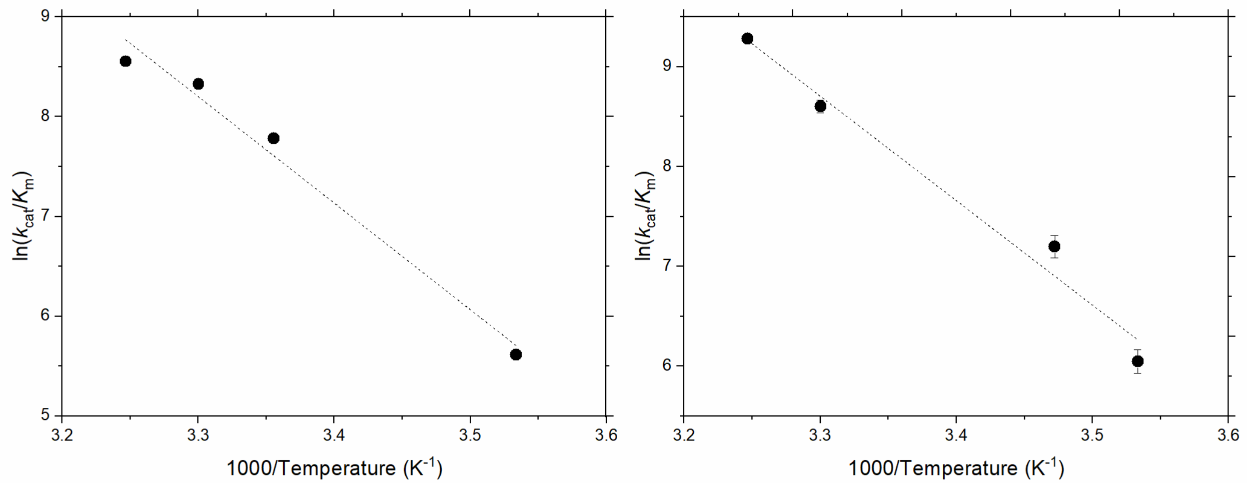

Figure S5. Arrhenius fits (Equation 1) for temperature dependent catalytic data for deglycosylation of enzyme using phosphate (left) and fructose (right). Experimental data are represented as averages (circles) with corresponding standard deviations (N = 3) in the form of error bars and the obtained linear fit is shown as dashed line. The correlation coefficients of the fits here are 0.97 (left) and 0.96 (right) respectively. The reactions were performed in the range of 10 – 35°C, while refer to Table S1 for detailed reaction composition. And the associated derived parameters from the fits are presented in Table 1.

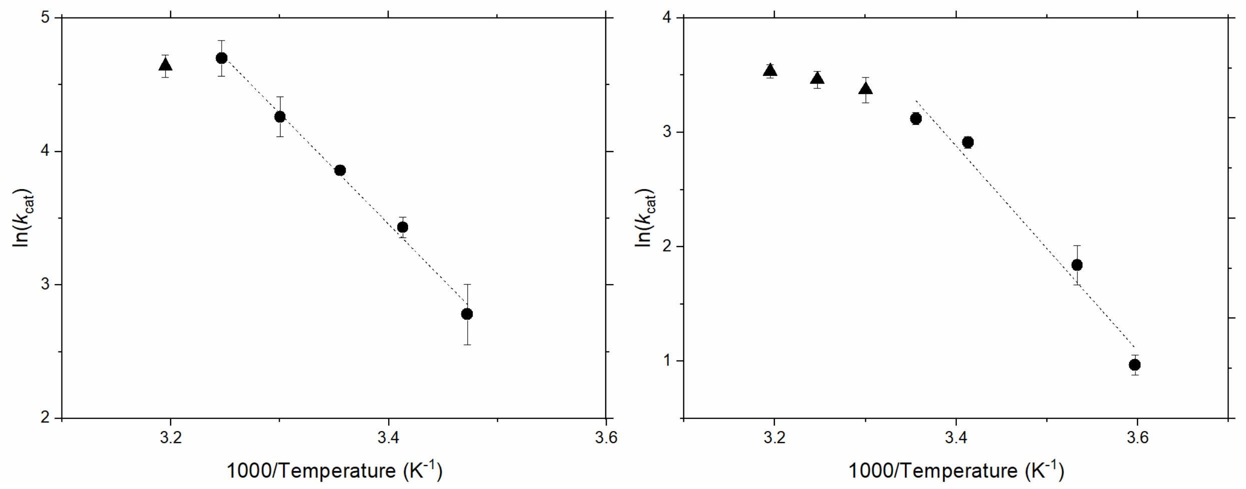

Figure S6. Arrhenius fits (Equation 1) for temperature dependent arsenolysis reactions using sucrose (left) and Glc1P (right) as glucosyl donors. Experimental data are represented as averages (circles) with corresponding standard deviations (N = 3) in the form of error bars and the obtained linear fit is shown as a dashed line. The circles denote linearly fitted points while triangles denote outliers of the fit as the fitted region is narrower than the overall data set. The correlation coefficients of the fits here are 0.99 (left) and 0.95 (right) respectively. The reactions were performed in the range of 5 – 40°C, while refer to Table S1 for detailed reaction composition. And the associated derived parameters from the fits are presented in Table 1.

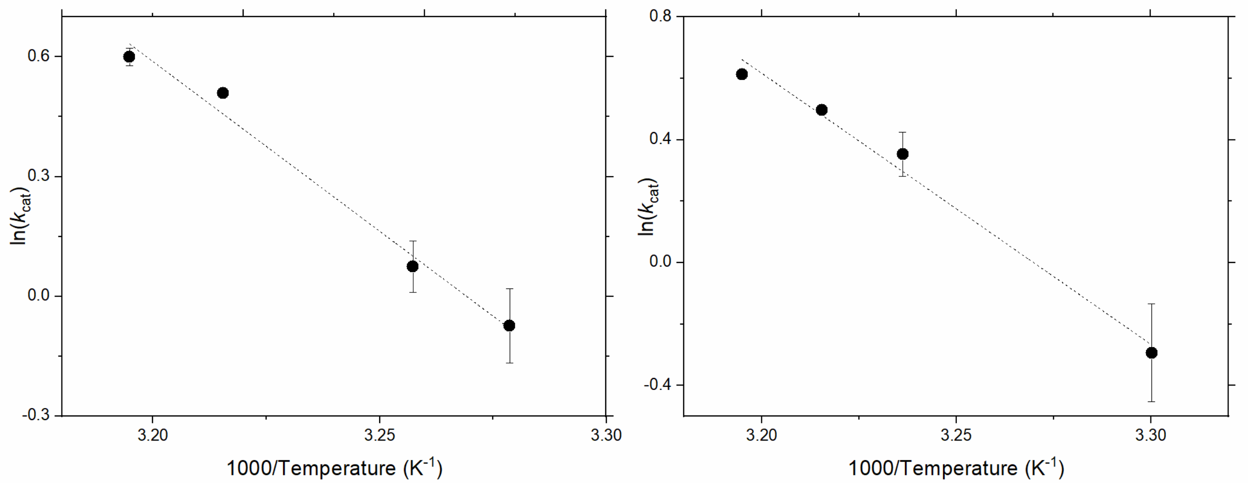

Figure S7. Arrhenius fits (Equation 1) for temperature dependent data of hydrolysis reactions using sucrose (left) and Glc1P (right) as glucosyl donors. Experimental data are represented as averages (circles) with corresponding standard deviations (N = 3) in the form of error bars and the obtained linear fit is shown as a dashed line. The correlation coefficients of the fits here are 0.97 (left) and 0.98 (right) respectively. The reactions were performed in the range of 30 – 40°C, while refer to Table S1 for detailed reaction composition. And the associated derived parameters from the fits are presented in Table 1.

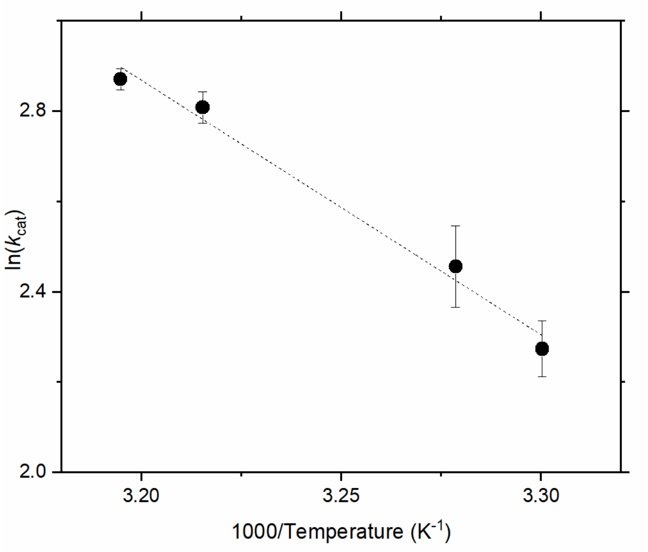

Figure S8. Arrhenius fit (Equation 1) for temperature dependent data of transglycosylation reaction to glycerol from sucrose assisted by sucrose phosphorylase. Experimental data are represented as averages (circles) with corresponding standard deviations (N = 3) in the form of error bars and the obtained linear fit is shown as a dashed line. The correlation coefficient of the fit is 0.98. The reactions were performed in the range of 30 − 40°C, while refer to Table S1 for detailed reaction composition. And the associated derived parameters from the fit are presented in Table 1.

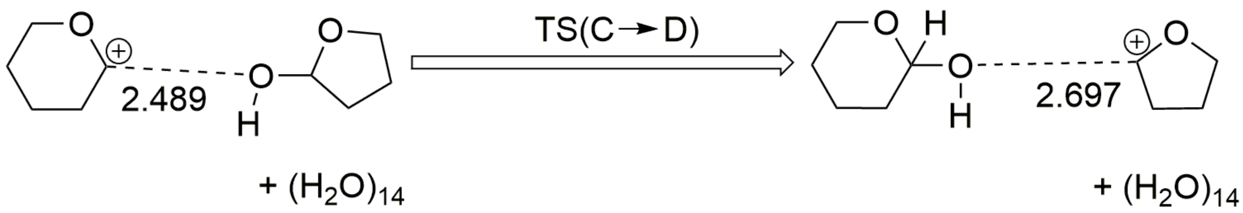

Figure S9. Oxocarbenium ion transition states for acid-catalyzed cleavage of sucrose. The Figure has been adapted from literature [8] which depicts the formation of a rather stable fructosyl oxocarbenium ion transition state by isomerization of glucosyl oxocarbenium transition state, explained in terms of a thermodynamic effect. Here, Int(C) denotes intermediate with protonated bridge oxygen of the glycosidic bond for the glucosyl-oxygen cleavage and Int(D) denotes protonated intermediate for the fructosyl-oxygen cleavage. All the distances are given in Å.

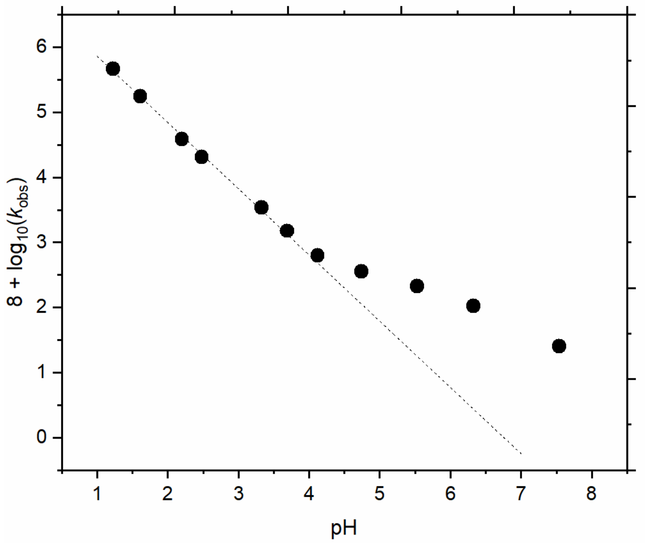

Figure S10. Estimation of the chemical (uncatalyzed) rate of hydrolysis of Glc1P via C1-O bond cleavage in the neutral (doubly protonated) molecular species at pH 7.0 [6]. The data shown are for the reaction of Glc1P at 82°C and are taken from literature (Bunton et al., 1958, J. Chem. Soc., 3588 – 3594. The Reactions of Organic Phosphates. Part II. The Hydrolysis of *α*-D-Glucose 1-(Dihydrogen Phosphate)). The Glc1P hydrolyzes via P-O bond cleavage at pH 7.0, so an extrapolation from the acidic region is necessary. Data in the pH range 1.23 – 3.33 are fitted to a straight line (dotted line). The correlation coefficient is 0.99, the slope is –1.02. The rate estimated at pH 7.0 and 82°C is 5.7×10^{-9} s^{-1}. Using the activation energy for the hydrolysis of the neutral Glc1P (E_a = 31 kcal mol^{-1}) [6] a rate of 3.0×10^{-12} s^{-1} is obtained.

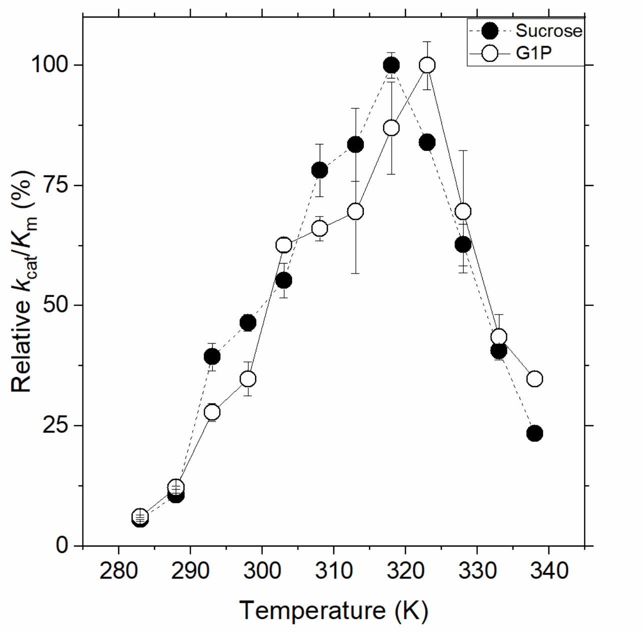

Figure S11. Temperature profiles of reactions assisted by sucrose phosphorylase with sucrose and Glc1P as glycosyl donors under substrate limiting conditions. The second substrate (50 mM phosphate, 50 mM fructose) was present at a saturating (Table S1). The glycosylation reactions with sucrose and Glc1P respectively, show a shift in T_m of around 5°C based on the glycosyl donor. In the plot, data associated standard deviations (N = 3) are shown in the form of error bars.

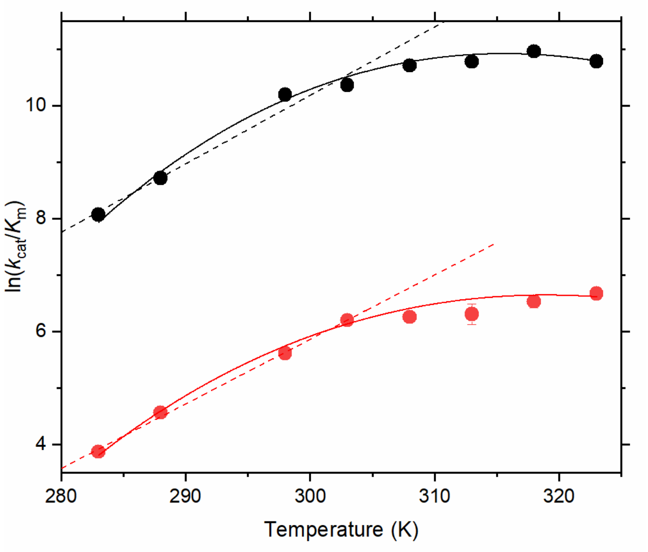

Figure S12. Fitting of Equation 3 (extended Arrhenius equation) to temperature dependent catalytic data of glycosylation of free enzyme by sucrose (black) and Glc1P (red) respectively for determination of ΔC_p of the enzyme during the course of the reaction. Compared to Figure S11, data used here are only up to 325 K because irreversible inactivation of the enzyme is not detectable in the time of the assay up to that temperature.

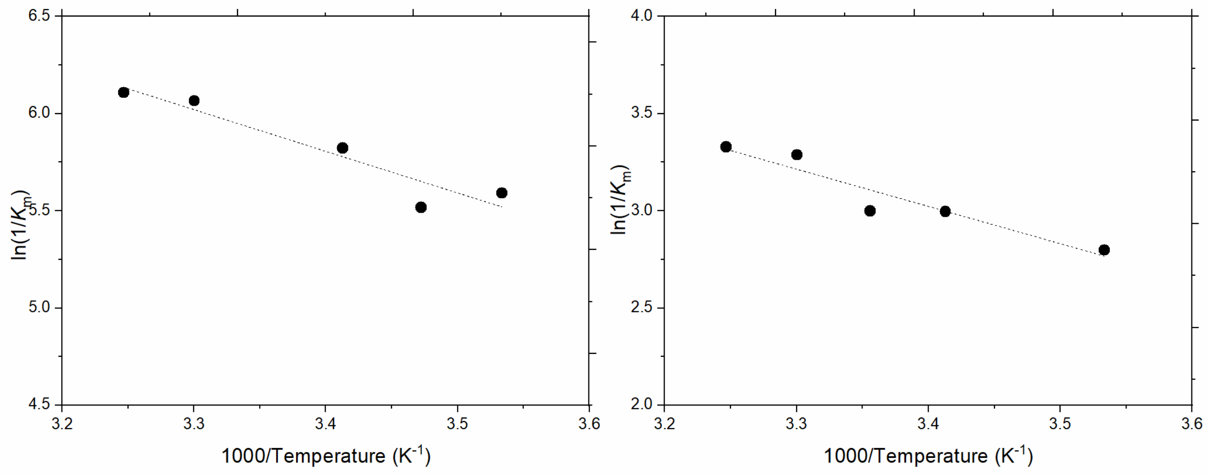

Figure S13. Arrhenius plots for temperature dependent K_m data for sucrose (left) and Glc1P (right). The $1/K_m$ values were fitted with correlation coefficient of 0.91. The K_m dependence on temperature here signifies favourable enzyme substrate binding with increasing temperature. The K_m values estimated from the plots at 30°C are 2.3 mM (sucrose) and ~3 × 10 mM (Glc1P).

S3. Supporting tables

Table S1. Compiled conditions for temperature-based characterization of respective reactions assisted by sucrose phosphorylase at pH 7.0 (Figure 1).

Reaction	To evaluate	Substrate(s) concentration	Enzyme (per mL reaction)	Analyte
Phosphorolysis	k_{cat}	50 mM sucrose;<br>50 mM phosphate	≤20°C: 0.6 μg<br>>20°C: 1.2 μg	Glc1P
	k_{cat}/K_m sucrose	0.5 – 2.0 mM [a,b]	<20°C: 0.3 μg<br>≥20°C: 0.4 μg	
	k_{cat}/K_m phosphate	2.0 – 8.0 mM [9,b]	<20°C: 0.7 μg<br>≥20°C: 1.0 μg	
Synthesis	k_{cat}	100 mM Glc1P;<br>50 mM fructose	≤20°C: 2.5 μg<br>>20°C: 5.0 μg	Phosphate
	k_{cat}/K_m Glc1P	5.0 – 20 mM [a]	<20°C: 2.0 μg<br>≥20°C: 3.5 μg	
	k_{cat}/K_m fructose	2.0 – 8.0 mM [10]	<20°C: 1.5 μg<br>≥20°C: 2.0 μg	
Hydrolysis	k_{cat}	50 mM sucrose	5.0 μg	Glucose
	k_{cat}	50 mM Glc1P		
Arsenolysis	k_{cat}	50 mM sucrose;<br>50 mM arsenate	<20°C: 0.5 μg<br>≥20°C: 1.0 μg	Glucose
	k_{cat}	200 mM Glc1P;<br>50 mM arsenate	<20°C: 1.8 μg<br>≥20°C: 3.5 μg	
Transfer reaction in the presence of glycerol	k_{cat}/K_m	50 mM sucrose;<br>2.0 M glycerol	3.5 μg	Glucose and fructose

[a]Based on Figure S4. [b]The second substrate was used at a constant and saturating concentration: phosphate (50 mM); sucrose (50 mM); fructose (50 mM); and Glc1P (100 mM).

Table S2. Energy levels plotted in the reaction coordinate diagram i.e., Figure 4.

Energy level	$\Delta G^{\ddagger\,a}$ in Figure 4 (kJ mol^{-1})	Equation used for calculation
enzyme + sucrose	0	Starting reference
enzyme-sucrose	−15	(4)
TS_1	+48	(5)
β-enzyme-glucosyl intermediate	−5.4	(8)
TS_2	+49	(5)
enzyme-Glc1P	−18	(4)
enzyme + Glc1P	−9.9	(6) and (7)
uncatalyzed TS (sucrose) [b]	+135	(5)
uncatalyzed TS (Glc1P) [b]	+131	(5)

[a]Values were estimated at 303 K. [b]k_{non} was used for uncatalyzed reactions.

Table S3. Parameters derived by fitting Equation 3 to temperature dependent catalytic data for enzyme glycosylation by sucrose and Glc1P respectively (Figure S12).

Substrate	ΔH (kJ mol^{-1})	ΔS (J K mol^{-1})	ΔC_p (kJ mol^{-1})	T_m (°C)	k_{cat}/K_m at T_m (M^{-1} s^{-1})
Sucrose	−14 ± 10	−200 ± 20	−4.1 ± 0.5	45	(48 ± 5.0) × 10^3
Glc1P	−17 ± 10	−241 ± 30	−3.2 ± 0.4	50	(1.2 ± 0.4) × 10^3

S5. Supporting video

Video S1. Loop movements in sucrose phosphorylase to accommodate binding of different glucosyl acceptors such as phosphate and fructose (provided separately).

S6. Supporting references

(1) Gudiminchi, R. K.; Nidetzky, B. Walking a Fine Line with Sucrose Phosphorylase: Efficient Single-Step Biocatalytic Production of L-Ascorbic Acid 2-Glucoside from Sucrose. *ChemBioChem.* **2017**, *18* (14), 1387–1390.

(2) Saheki, S.; Takeda, A.; Shimazu, T. Assay of Inorganic Phosphate in the Mild pH Range, Suitable for Measurement of Glycogen Phosphorylase Activity. *Anal. Biochem.* **1985**, *148* (2), 277–281.

(3) Klotzsch, H.; Bergmeyer, H.-U. D-fructose. In H.-U. Bergmeyer (Ed.), Methods of enzymatic analysis. Cambridge, MA*: Academic Press.* **1965**, 156–159.

(4) Klimacek, M.; Sigg, A.; Nidetzky, B. On the Donor Substrate Dependence of Group-Transfer Reactions by Hydrolytic Enzymes: Insight from Kinetic Analysis of Sucrose Phosphorylase-Catalyzed Transglycosylation. *Biotechnol. Bioeng.* **2020**, *117* (10), 2933–2943.

(5) Torres, A. P.; Oliveira, F. A. R.; Silva, C. L. M.; Fortuna, S. P. The Influence of pH on the Kinetics of Acid Hydrolysis of Sucrose. *J. Food Process Eng.* **1994**, *17* (2), 191–208.

(6) Bunton, C. A.; Llewellyn, D. R.; Oldham, K. G.; Vernon, C. A. 717. The Reactions of Organic Phosphates. Part II. The Hydrolysis of α-D-Glucose 1-(Dihydrogen Phosphate). *J. Chem. Soc. Perk Trans.* **1958**, 3588–3594.

(7) Schwarz, A.; Brecker, L.; Nidetzky, B. Acid–Base Catalysis in *Leuconostoc mesenteroides* Sucrose Phosphorylase Probed by Site-Directed Mutagenesis and Detailed Kinetic Comparison of Wild-Type and Glu 237 → Gln Mutant Enzymes . *Biochem. J.* **2007**, *403* (3), 441–449.

(8) Yamabe, S.; Guan, W.; Sakaki, S. Three Competitive Transition States at the Glycosidic

Bond of Sucrose in Its Acid-Catalyzed Hydrolysis. *J. Org. Chem.* **2013**, *78* (6), 2527–2533.

(9) Goedl, C.; Schwarz, A.; Minani, A.; Nidetzky, B. Recombinant Sucrose Phosphorylase from *Leuconostoc mesenteroides*: Characterization, Kinetic Studies of Transglucosylation, and Application of Immobilised Enzyme for Production of α-D-Glucose 1-Phosphate. *J. Biotechnol.* **2007**, *129* (1), 77–86.

(10) Wildberger, P.; Luley-Goedl, C.; Nidetzky, B. Aromatic Interactions at the Catalytic Subsite of Sucrose Phosphorylase: Their Roles in Enzymatic Glucosyl Transfer Probed with Phe52 → Ala and Phe52 → Asn Mutants. *FEBS Lett.* **2011**, *585* (3), 499–504.
